# Correlation Between Neutrophil–Lymphocyte Ratio and Inhospital Cardiac Events in Geriatric Patients With Hip Fractures

**DOI:** 10.1155/mi/5587265

**Published:** 2024-12-17

**Authors:** Miaomiao Yu, Zhen Cui, Ying Bai

**Affiliations:** Intensive Care Unit, Beijing Jishuitan Hospital Affiliated to Capital Medical University, Beijing 100000, China

**Keywords:** elderly, hip fracture, MACE, NLR

## Abstract

**Background:** The novel inflammatory biomarker known as the neutrophil–lymphocyte ratio (NLR) has shown great potential in predicting and prognosing many diseases. However, its correlation with postoperative inhospital major adverse cardiac events (MACEs) in geriatric patients with hip fractures remains unclear. This study investigated the relationship between NLR and postoperative inhospital MACEs among geriatric patients with hip fractures.

**Methods:** We enrolled geriatric patients with hip fractures who were hospitalized in the Department of Geriatric Traumatology and Orthopedics, Beijing Jishuitan Hospital, Capital Medical University, between January 2023 and November 2023. After surgery, the patients were transferred to the intensive care unit (ICU) for postoperative monitoring and treatment. Patients were assigned to the MACE or non-MACE group based on the occurrence of MACEs after surgery during their hospital stay. Clinical data were retrospectively analyzed.

**Results:** In all, 216 patients were recruited into the study: 34 in the MACE group and 182 in the non-MACE group. Univariate and multivariate analyses revealed that a medical history of stroke (odds ratio (OR) = 2.66, 95% confidence interval (CI) = 1.18–6.01; *p*=0.018) and elevated preoperative NLR (OR = 1.09, 95% CI = 1.03–1.17; *p*=0.005) were significant risk factors for postoperative inhospital MACEs. The area under the curve (AUC) of preoperative NLR-predicted MACEs was 0.65 (0.55–0.75). Patients with a preoperative NLR <6.49 were less likely to experience inhospital MACEs, demonstrating a sensitivity of 61.8% and specificity of 64.8%.

**Conclusion:** Elevated preoperative NLR is an independent risk factor for postoperative inhospital MACEs in geriatric patients with hip fractures.

## 1. Introduction

The incidence of hip fractures worldwide reached 14.2 million in 2019, and the age-standardized incidence rate was 182.5/100,000 people [1]. By 2050, the annual global occurrence of hip fractures is predicted to increase to approximately 7–21 million [2]. Hip fractures significantly impact patients' health and quality of life, imposing a high financial burden on both the patients' families and society. These fractures mostly affect geriatric patients with an average age >80 years [3]. Surgery is the primary treatment for hip fractures; however, the 1-year postoperative mortality rate is only 17.5% [4]. Increased mortality following hip fracture surgery is associated with postoperative complications. Studies indicate that the incidence rate of cardiac events within 30 days of hip fracture is as high as 22.2%, ranking as the second most common cause of death following hip fractures [5, 6]. Hip fracture itself serves as a significant indicator of cardiac events [7], and geriatric patients with hip fractures usually have complications, including hypertension, diabetes, coronary heart disease, hyperlipidemia, and other underlying diseases. Data from the National Health and Nutritional Examination Survey (NHANES) suggest that age, race, smoking, and alcohol consumption, combined with diabetes and osteoporosis, are risk factors for hip fracture in elderly patients [8], and these conditions collectively pose a high risk for cardiac events. Zhang et al. [9] suggested that low dietary choline intake is associated with osteoporosis in elderly patients. Another study showed that low choline intake leads to a high inflammatory state in healthy adults [10]. An increase in the systemic immune-inflammation index, another indicator of the body's inflammatory state, has also been found to be associated with osteoporosis [11]. Moreover, elderly people with hip fractures are in a state of high inflammation. Factors, such as blood loss, intraoperative and postoperative hypotension, and the release of inflammatory factors, are likely to lead to postoperative cardiac events [12].

A novel inflammatory biomarker, the neutrophil–lymphocyte ratio (NLR), is often used to assess systemic inflammation [13]. Research has shown an association between a high NLR and postoperative delirium, fatigue, pneumonia, and other adverse events, including increased mortality in geriatric patients with hip fractures [13–17]. A study showed that postoperative NLR can reflect the degree of surgery-related trauma in young and middle-aged patients with humeral shaft fractures with high sensitivity and specificity [18]. Similarly, in elderly patients with subtrochanteric fractures, NLR was closely related to the degree of aseptic inflammation after surgery. Thus, NLR can provide a reference for the development of individualized surgical methods for patients [19]. Early identification and management of high-risk patients positively impacts prognosis. Studies have indicated that preoperative cardiovascular disease, high-sensitive troponin I (hs-TNI), low left ventricular ejection fraction (LVEF), and high N-terminal pro-brain natriuretic peptide (NT-proBNP) might be independent risk factors for major adverse cardiac events (MACEs) in elderly patients with hip fracture [5, 20, 21]. In other studies, elevated NLR was found to have a close relationship with the occurrence of MACEs in acute myocardial infarction [22], chronic heart failure [23], and atrial fibrillation (AF) [24]. However, the relationship between NLR and postoperative inhospital MACEs in geriatric patients with hip fractures remains unclear. Therefore, the goal of this research was to investigate the relationship between NLR and postoperative MACEs and its predictive effect on postoperative MACEs during hospitalization in geriatric patients with hip fractures.

## 2. Materials and Methods

### 2.1. Patients

The clinical data of geriatric patients with hip fractures hospitalized at the Department of Geriatric Traumatology and Orthopedics, Beijing Jishuitan Hospital, Capital Medical University, between January 2023 and November 2023 were retrospectively analyzed. The inclusion criteria were individuals over 65 years of age; a diagnosis of unilateral femoral neck, intertrochanteric, or subtrochanteric fracture based on clinical symptoms, signs, and imaging examination; surgical treatment for hip fracture; intensive care unit (ICU) admission for postoperative monitoring and treatment and evaluation by the surgeon and anesthesiologist; and trauma resulting from mild violence (such as falling, twisting, lifting heavy objects, etc.). The exclusion criteria were an expected survival time of <24 h, undergoing hip fracture revision surgery, pathological fracture, missing or incomplete information, and coinfection or hormonal drug use before surgery. Based on whether MACEs occurred in the hospital after surgery, patients were classified into the MACE and non-MACE groups. Postoperative MACEs refer to new-onset stroke, acute coronary syndrome, myocardial infarction, acute exacerbation of heart failure, and new-onset AF during hospitalization after surgery [7].

### 2.2. Data Collection

Patient data, including demographic data (sex, age, and body mass index (BMI)), medical history (renal insufficiency, diabetes, coronary artery disease, hypertension, chronic cardiac insufficiency, stroke, and chronic obstructive pulmonary disease (COPD), etc.), surgical data (type of fracture, surgical procedure, anesthesia, intraoperative hemorrhage, and American Society of Anesthesiologists (ASA) grading), and laboratory information (hematocrit (HCT) on admission and preoperatively, NLR on admission and preoperatively, albumin on admission, NT-proBNP on admission and preoperatively, hs-TNI on admission and preoperatively, and preoperative LVEF), were collected from a clinical electronic health record system.

### 2.3. Statistical Analysis

All data were analyzed using Predictive Analytics Software (PASW) Statistics version 18.0.0 (Statistical Product and Service Solutions (SPSS) Inc., Chicago, IL, USA). Means and standard deviations were employed for continuous variables exhibiting a normal distribution, whereas medians and interquartile ranges were used for nonnormally distributed continuous data. Categorical variables are presented as percentages and frequencies. Two-sided tests were used, with *p* < 0.05 representing statistical significance. Continuous variables were compared using the student *t*-test or Wilcoxon rank-sum test, and the *χ*^2^ or Fisher exact test was employed for categorical variables. Univariate logistic regression analysis was performed on the clinical data to identify the risk factors for MACEs during the hospital stay following surgery. Variables that were significant (*p* < 0.10) in the univariate analyses were included in the multivariate analyses to determine the independent risk factors for MACEs. The predictive efficacy of NLR for MACEs was assessed using a receiver operating characteristic (ROC) curve.

## 3. Results

### 3.1. Clinic Characteristics

**Figure 1:**
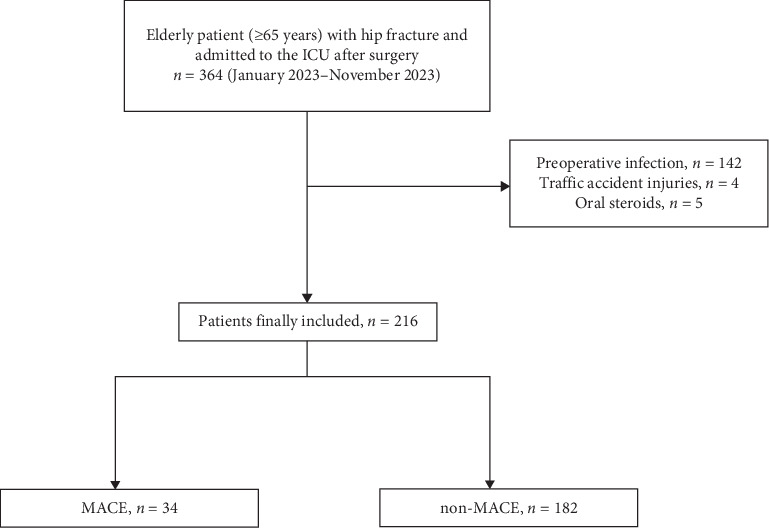
Flowchart of the study. ICU, intensive care unit; MACE, major adverse cardiac event.

Overall, 364 patients were included; 148 patients were excluded, comprising 142 cases of preoperative coinfection, 4 cases of injuries caused by violent factors, such as car accidents, and 5 cases of long-term preoperative steroid therapy. Among the 216 patients, 34 were enrolled in the MACE group and 182 were enrolled in the non-MACE group (Figure 1); 85 were male and 131 were female. The incidence of inhospital MACEs after hip fracture was 15.7%. The average age in the MACE group was 85.0 ± 6.6 years, compared to 84.5 ± 7.9 years in the non-MACE group. The main type of fracture was intertrochanteric (56.9%), followed by femoral neck (39.4%). No statistical differences were observed between the two groups concerning baseline characteristics such as age, sex, Acute Physiology and Chronic Health Evaluation II (APACHE II) score, BMI, medical history of renal insufficiency, hypertension, diabetes, COPD, cardiac insufficiency, coronary artery disease, fracture type, anesthesia method, surgery type, ASA grade, intraoperative blood loss, HCT, hs-TNI, NT-proBNP, albumin, NLR on admission, preoperative HCT, or preoperative LVEF. Significantly higher rates of stroke, lower rates of AF, increased preoperative NLR, higher inhospital mortality, and longer ICU and hospital stays were found in the MACE group (Table 1).

### 3.2. Independent Risk Factors for MACEs

According to the findings of the univariate regression analysis, a medical history of AF was a protective factor for MACEs (*p* < 0.05). In contrast, a medical history of stroke and preoperative NLR elevation were risk factors for MACEs (*p* < 0.05; Table 2).

Multivariate analysis revealed that an elevated preoperative NLR (odds ratio (OR) = 1.09, 95% confidence interval (CI) = 1.03–1.17, *p* < 0.05) and a medical history of stroke (OR = 2.66, 95% CI = 1.18–6.01, *p* < 0.05) were independent risk factors for postoperative inhospital MACEs (*p* < 0.05; Table 3).

### 3.3. Predictive Ability of the NLR for Inhospital MACEs

**Figure 2:**
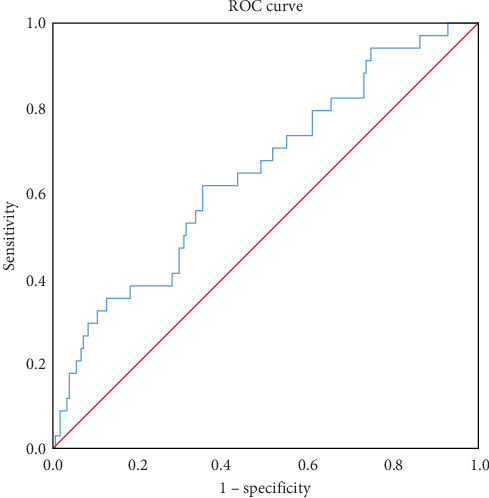
ROC curve for the predictive ability of preoperative NLR for MACE. MACE, major adverse cardiac event; NLR, neutrophil–lymphocyte ratio; ROC, receiver operating characteristic.

The ROC curve revealed the predictive power of NLR for inhospital MACEs (Figure 2). The area under the curve (AUC) was 0.65 (0.55–0.75). The best cutoff value was 6.49, with a sensitivity and specificity of 61.8% and 64.8%, respectively.

## 4. Discussion

Of the 216 patients enrolled in this study, 34 (15.1%) experienced postoperative MACEs in the hospital. Hsu et al. [7] revealed that 13.3% of geriatric hip fractures experienced MACEs within 1 year; the incidence of MACEs in 1 month after hip fracture in geriatric patients was 16.4% in another study [20]. There may be several reasons for the different rates of MACEs, and different study populations may be one of these reasons. This study enrolled patients admitted to the ICU for monitoring and treatment after surgery. These patients often have more complex medical histories, worse nutritional status, and severe abnormalities on preoperative laboratory examinations; therefore, the incidence of postoperative MACEs is higher. Additionally, we focused on MACEs that occurred in the hospital, and the follow-up time was relatively short, contributing to the different incidences of MACEs monitored.

Geriatric patients with hip fractures who experience postoperative inhospital MACEs have significantly increased incidence and mortality rates. Therefore, it is crucial to identify high-risk patients. A previous study showed that old age, preoperative LVEF <50%, and preoperative hs-TNI >6.5 pg/ml were independent risk factors for postoperative MACEs [9]. Another study reported that patients with a history of cardiovascular disease had a 2.86-fold increased risk of MACEs [21]. The study results of Chao et al. [25] also showed that 93.7% of cardiovascular events following hip fractures occurred in geriatric patients with a history of cardiovascular diseases. Similarly, our study found that a medical history of stroke was independently related to postoperative inhospital MACEs; however, a prior history of AF and coronary heart disease was not statistically different between the two groups. Another observational study involving 450 patients found that preoperative NT-proBNP >600 pg/ml could predict postoperative cardiovascular complications [26]. Owing to the different study designs and included populations, the risk factors for MACEs after hip fractures in geriatric patients have been inconsistent in different studies. Our study found no correlation between MACEs and age, preoperative low LVEF, preoperative hs-TNI, or NT-proBNP. Similar to a previous study [20], different surgery types were not related to the occurrence of MACEs in our study.

The mechanism of MACEs after hip fracture is not completely clear and may be related to factors such as elevated blood calcium levels after fracture, common risk factors for cardiovascular events and fracture (e.g., lack of exercise, advanced age, and increased inflammation), and the administration of nonsteroidal anti-inflammatory drugs for analgesia after fracture [7]. It was found that interleukin (IL)-6 and keratinocyte chemoattractant levels were elevated in mice after hip fracture. Inflammatory markers, such as high mobility group box 1 protein (HMGB1), toll-like receptors 2 and 4 (TLR2/4), tumor necrosis factor (TNF), IL1b, and nucleotide-binding oligomerization domain, leucine-rich repeat, and pyrin domain-containing 3 (NLRP3), were also found to increase in the myocardium after hip fracture, indicating that the inflammatory response may be one of the causes of secondary myocardial injury after hip fracture [27].

As a simple and easily accessible biomarker of inflammation, NLR has been widely used in many fields, including cardiovascular diseases. In patients with heart failure, an elevated NLR is an indicator of high morbidity and mortality and other unfavorable outcomes such as rehospitalization and cardiovascular events [28, 29]. A lower NLR is observed in inhospital surviving acute myocardial infarction patients [30], and an elevated NLR after percutaneous coronary intervention (PCI) indicates an association with a larger infarct area [31]. In patients with acute myocardial infarction, NLR was also found to be an independent risk indicator for acute heart failure and arrhythmia but was not linked to the occurrence of heart failure and arrhythmia within 1 year [32]. A meta-analysis showed that elevated NLR on admission, postprocedure, or during the procedure is associated with the risk of AF occurrence and recurrence [33]. In patients with AF, high NLR levels are associated with bleeding incidents, stroke/systemic embolism, all-cause mortality, and MACEs [24, 34]. NLR was found to be significantly associated with adverse outcomes in an analysis of data from several large trials [35]. Although many studies have explored the correlation between NLR and various diseases and prognoses, there are no standardized criteria for the normal NLR range.

To the best of our knowledge, this study is the first to explore the correlation between NLR and postoperative inhospital MACEs in geriatric patients with hip fractures. Our results indicated that a preoperative increased NLR was an independent risk factor for inhospital MACEs in this population. The OR value after multivariate analysis was 1.09 (1.03–1.17). The AUC of MACEs predicted by the preoperative NLR was 0.65, and the optimal cutoff value of the NLR was 6.49. NLR offers moderate predictability for the occurrence of MACEs but is not an ideal biomarker. As NLR is an inflammatory biomarker, the association and predictive value of other inflammatory markers in MACEs can be explored to determine the most accurate and effective indicators. It is challenging to predict MACEs using only NLR; therefore, a prediction model that includes other risk factors is required for accurate prediction. Moreover, the association between NLR and other complications after hip fracture in elderly patients should be explored.

This study had several limitations. First, it was a single-center study and only enrolled geriatric patients requiring intensive care after hip fracture surgery. Therefore, it may be necessary to expand the scope of this study further to obtain more extrapolated conclusions. Second, this study mainly evaluated the correlation between preoperative NLR and postoperative MACEs in geriatric patients with hip fractures during their hospital stay; the long-term prognosis requires further verification. Finally, because this study was retrospective, data on other inflammatory markers were unavailable. Therefore, the correlation between other inflammatory biomarkers and the occurrence of MACEs after hip fracture in geriatric patients needs further exploration.

## 5. Conclusion

An elevated NLR is a significant independent risk factor and predictor of postoperative inhospital MACEs in geriatric patients with hip fractures, and the best cutoff value was 6.49. Individuals with a preoperatively elevated NLR (>6.49) should be given more attention and monitored closely, and vigilance should be maintained regarding the occurrence of MACEs. NLR can be included as an independent risk factor in MACE forecasting models or used in combination with other indicators to improve forecasting accuracy. NLR can also help doctors stratify the risk level of patients, which can aid clinicians in focusing on high-risk patients and rationally allocating medical resources. However, its predictive efficacy is not high; therefore, further exploration of effective indicators and models that can predict MACEs is necessary.

## Figures and Tables

**Table 1:** Characteristics of patients in the MACE and non-MACE groups.

Variables	Total (*n* = 216) *n* (%) or mean ± SD or median (IQR)	Non-MACE (*n* = 182) *n* (%) or mean ± SD or median (IQR)	MACE (*n* = 34) *n* (%) or mean ± SD or median (IQR)	*p* Value
Male	85 (39.4)	70 (38.5)	15 (44.1)	0.535
Age (years)	84.5 ± 7.7	84.5 ± 7.9	85.0 ± 6.6	0.72
BMI (kg/m^2^)	22.0 (19.9–24.5)	22.0 (19.9–24.5)	22.0 (20.0–24.4)	0.774
APACHE II	14.0 (12.0–17.0)	14.0 (12.0–17.0)	14.0 (12.2–17.8)	0.307
Medical history	—	—	—	—
Hypertension	135 (62.5)	116 (63.7)	19 (55.9)	0.385
Diabetes	74 (34.3)	67 (36.8)	7 (20.6)	0.067
Coronary artery disease	85 (39.4)	72 (39.6)	13 (38.2)	0.885
COPD	7 (3.2)	6 (3.3)	1 (2.9)	1
Stroke	54 (25.0)	40 (22)	14 (41.2)	0.018
AF	41 (19.0)	39 (21.4)	2 (5.9)	0.034
Cardiac insufficiency	29 (13.4)	22 (12.1)	7 (20.6)	0.18
Renal insufficiency	27 (12.5)	21 (11.5)	6 (17.6)	0.394
Fracture location	—	—	—	0.493
Intertrochanteric	123 (56.9)	101 (55.5)	22 (64.7)	—
Subtrochanteric	8 (3.7)	8 (4.4)	0 (0)	—
Femoral neck	85 (39.4)	73 (40.1)	12 (35.3)	—
Surgery type	—	—	—	1
Total hip arthroplasty	3 (1.6)	3 (1.4)	0 (0)	—
Partial hip arthroplasty	70 (32.4)	59 (32.4)	11 (32.4)	—
Internal fixation	143 (66.2)	120 (65.9)	23 (67.9)	—
Anesthesia method	—	—	—	0.781
Intraspinal anesthesia	188 (87.0)	159 (87.4)	29 (85.3)	—
General anesthesia	28 (13.0)	23 (12.6)	5 (14.7)	—
ASA (*n*)	—	—	—	0.732
II	73 (33.8)	63 (34.6)	10 (29.4)	—
III	142 (65.7)	118 (64.8)	24 (70.6)	—
IV	1 (0.5)	1 (0.5)	0 (0)	—
Blood loss (ml)	200.0 (100.0–200.0)	200.0 (100.0–200.0)	200.0 (100.0–200.0)	0.245
HCT on admission	32.7 ± 6.4	32.5 ± 6.2	34.0 ± 7.2	0.226
hs-TNI on admission (pg/ml)	11.1 (6.7–22.9)	11.1 (6.5–23.6)	12.3 (8.9–19.2)	0.43
NT-proBNP on admission (pg/ml)	649.2 (298.5–1663.0)	637.3 (296.4–1704.0)	791.4 (317.6–1571.0)	0.838
HCT before surgery	30.7 ± 5.4	30.5 ± 5.3	31.4 ± 5.9	0.371
Albumin on admission (g/L)	37.5 ± 3.6	37.4 ± 3.7	38.2 ± 2.9	0.24
NLR on admission	7.5 (5.3–11.0)	7.2 (5.3–11.0)	8.2 (5.4–11.6)	0.501
NLR before surgery	5.7 (4.2–7.9)	5.4 (3.9–7.6)	6.9 (4.7–14.0)	0.006
LVEF on admission ≤50%	4.6	4.9	2.9)	1
Survival	206 (95.4)	178 (97.8)	28 (82.4)	0.001
Length of ICU (days)	2.0 (1.0–3.0)	2.0 (1.0–3.0)	3.0 (2.0–5.0)	<0.001
Length of hospital (days)	6.0 (5.0–9.0)	6.0 (5.0–9.0)	7.5 (6.0–11.8)	0.049

Abbreviations: AF, atrial fibrillation; APACHE II, Acute Physiology and Chronic Health Evaluation II; ASA, American Society of Anesthesiologists; BMI, body mass index; COPD, chronic obstructive pulmonary disease; HCT, hematocrit; hs-TNI, high-sensitive troponin I; IQR, interquartile range; LVEF, left ventricular ejection fraction; MACE, major advanced cardiovascular event; NLR, neutrophil–lymphocyte ratio; NT-proBNP, N-terminal pro-brain natriuretic peptide; SD, standard deviation.

**Table 2:** Univariate logistic regression analysis for the association between NLR and MACEs.

Variable	Univariate logistic analysis OR (95% CI)	*p*-Value
Male	1.26 (0.60–2.65)	0.536
Age >80	0.58 (0.21–1.59)	0.282
BMI (kg/m)^2^		
<18.5	1 (Ref.)	—
18.5–24.9	1.25 (0.4–3.93)	0.703
≥25	1.04 (0.28–3.92)	0.952
APACHE II	1.07 (0.97–1.17)	0.164
Hypertension	0.72 (0.34–1.51)	0.387
Diabetes	0.44 (0.18–1.08)	**0.073**
Coronary artery disease	0.95 (0.45–2.01)	0.885
COPD	0.89 (0.1–7.62)	0.914
Stroke	2.48 (1.15–5.36)	**0.02**
AF	0.23 (0.05–1)	**0.05**
Cardiac insufficiency	1.89 (0.73–4.84)	0.188
Renal insufficiency	1.64 (0.61–4.43)	0.327
Fracture location		
Trochanteric	1 (Ref.)	—
Subtrochanteric	0 (0–Inf)	1
Femoral neck	0.75 (0.35–1.62)	0.471
Surgery type		
Total hip arthroplasty	1 (Ref.)	—
Partial hip arthroplasty	0 (0–Inf)	0.999
Internal fixation	0 (0–Inf)	0.999
Anesthesia method, *n* (%)		
Intraspinal anesthesia	1 (Ref.)	—
General anesthesia	1.19 (0.42–3.39)	0.742
ASA		
II	1 (Ref.)	—
III	1.28 (0.58–2.85)	0.543
IV	0 (0–Inf)	1
Blood loss (ml)	1 (0.99–1)	0.16
HCT before surgery	1.04 (0.98–1.1)	0.226
hs-TNI on admission (pg/ml)	1 (1–1.01)	0.433
NT-proBNP on admission (pg/ml)	1 (1–1)	0.353
Albumin on admission (g/L)	1.06 (0.96–1.18)	0.239
LVEF ≤50% on admission	0.58 (0.07–4.75)	0.614
NLR on admission	1.01 (0.96–1.06)	0.787
NLR before surgery	1.1 (1.03–1.16)	**0.003**

*Note:* Bold values indicate the variables of *p* < 0.1 and were included in the multivariate analyses.

Abbreviations: AF, atrial fibrillation; APACHE II, Acute Physiology and Chronic Health Evaluation II; ASA, American Society of Anesthesiologists; BMI, body mass index; CI, confidence interval; COPD, chronic obstructive pulmonary disease; HCT, hematocrit; hs-TNI, high-sensitive troponin I; Inf, infimum; LVEF, left ventricular ejection fraction; MACE, major advanced cardiovascular event; NLR, neutrophil–lymphocyte ratio; NT-proBNP, N-terminal pro-brain natriuretic peptide; OR, odds ratio; Ref., reference.

**Table 3:** Multivariable logistic regression analysis for the association between NLR and MACEs.

Variable	Multivariable logistic analysis OR (95% CI)	*p* Value
Diabetes	0.46 (0.18–1.18)	0.105
Stroke	2.66 (1.18–6.01)	**0.018**
AF	0.25 (0.06–1.1)	0.067
NLR before surgery	1.09 (1.03–1.17)	**0.005**

*Note:* Bold represents that they are statistically significant (*p* < 0.05).

Abbreviations: AF, atrial fibrillation; CI, confidence interval; MACE, major advanced cardiovascular event; NLR, neutrophil–lymphocyte ratio; OR, odds ratio.

## Data Availability

The data for this paper can be obtained by contacting the corresponding author.
